# Circadian dysregulation of leptin and its role in skin inflammation and obesity: an integrative perspective

**DOI:** 10.1007/s12020-026-04809-y

**Published:** 2026-10-07

**Authors:** Luiza A. Altenfelder, Ellen M. S. Xerfan, Sergio Tufik, Monica L. Andersen

**Affiliations:** 1 Escola Paulista de Medicina, Universidade Federal de São Paulo (UNIFESP), São Paulo, Brazil; 2 Departamento de Psicobiologia, Universidade Federal de São Paulo (UNIFESP), São Paulo, Brazil; 3 Instituto do Sono, Associação Fundo de Incentivo à Pesquisa (AFIP), São Paulo, Brazil

**Keywords:** Sleep, Circadian rhythm, Circadian misalignment, Obesity, Skin inflammation, Meal timing

## Abstract

**Purpose:**

Leptin is an adipokine that regulates energy balance and is commonly linked to obesity. It exhibits a circadian rhythm that can be disrupted by variations in eating times and sleep disorders. This brief review explored how leptin can modulate appetite and tissue repair; and assessed whether dysregulated serum levels may activate pro-inflammatory pathways contributing to inflammatory dermatoses, particularly in the context of obesity.

**Methods:**

Current evidence on the interconnection between leptin rhythmicity, obesity, sleep disruption, meal timing, oxidative stress, and inflammatory dermatoses was reviewed and synthesized, with an emphasis on studies relevant to the proposed circadian, metabolic and cutaneous axis.

**Results:**

Available evidence indicated that irregular or late meal timing can alter leptin rhythmicity, while sleep disruption is associated with changes in leptin secretion and inflammatory mediators. In obesity, persistent hyperleptinemia may coexist with central leptin resistance and preserved peripheral pro-inflammatory signaling. Experimental and clinical findings support associations among leptin dysregulation, oxidative stress, reduced antioxidant capacity, and inflammatory pathways relevant to dermatoses related to obesity, particularly psoriasis. However, direct evidence establishing a causal relationship between circadian leptin dysregulation and skin inflammation remains limited.

**Conclusions:**

Circadian leptin misalignment may represent a plausible upstream pathway linking altered meal timing, sleep disruption, leptin resistance, oxidative stress, and immune activation to obesity-associated inflammatory dermatoses. Chronobiological interventions, particularly time-restricted feeding combined with adequate sleep, warrant further investigation for their potential to improve leptin regulation and attenuate skin inflammation. Much of the current evidence remains associative, and further mechanistic and interventional studies are needed to test this hypothesis.

## Introduction

**Fig. 1:**
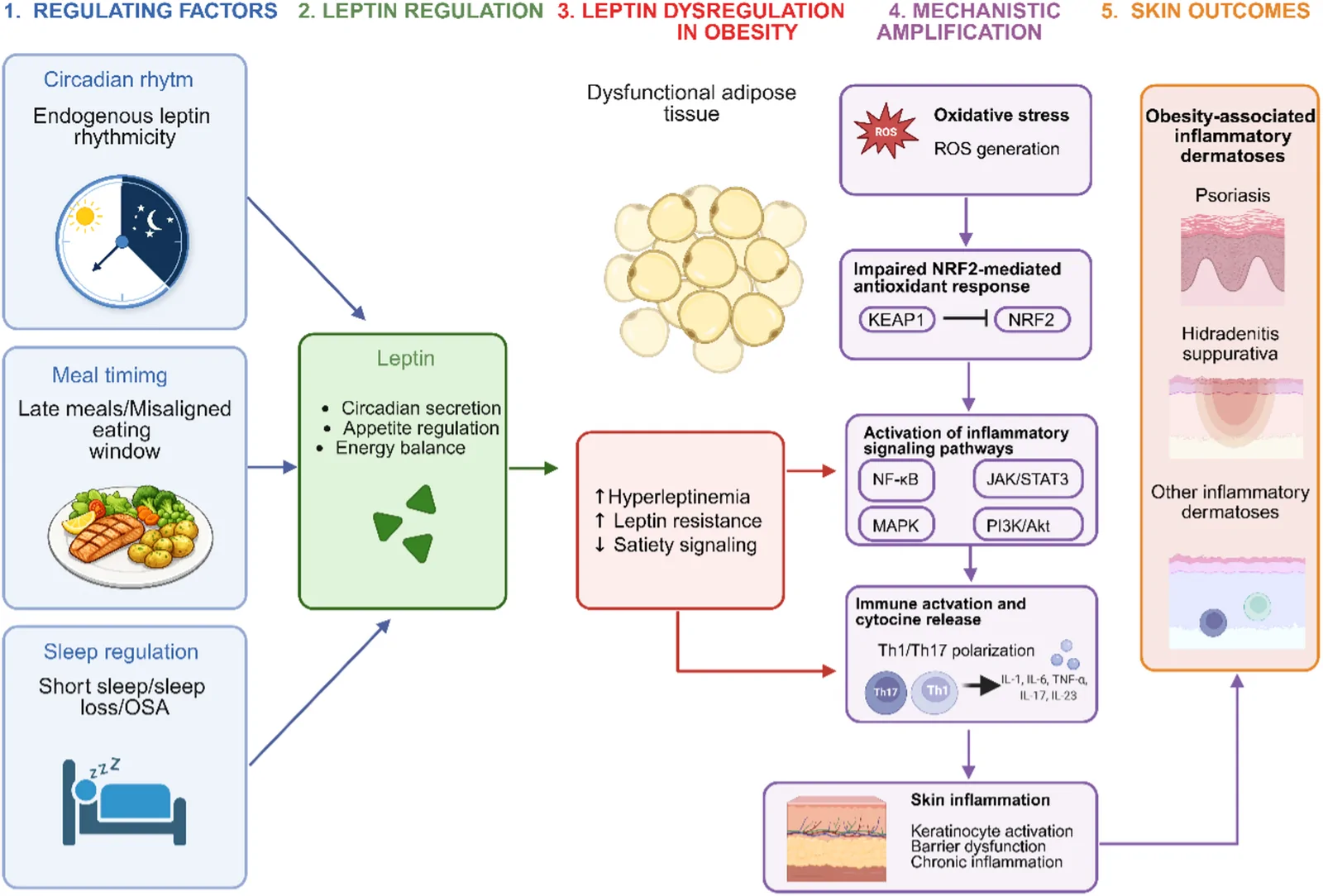
Proposed role of circadian, metabolic, and cutaneous pathways linking leptin dysregulation to skin inflammation in obesity. Circadian rhythm, meal timing, and sleep regulation may influence leptin secretion, appetite regulation, and energy balance. In obesity, dysfunctional adipose tissue may promote hyperleptinemia, leptin resistance, and impaired satiety signaling. These changes may increase oxidative stress, ROS generation, impaired antioxidant responses mediated by NRF2, activation of inflammatory signaling pathways, Th1/Th17 polarization, cytokine release, and chronic skin inflammation. These mechanisms may contribute to inflammatory dermatoses associated with obesity, particularly psoriasis and hidradenitis suppurativa. Legends: ROS, reactive oxygen species; KEAP1, Kelch-like ECH-associated protein 1; NRF2, nuclear factor erythroid 2 related factor 2; NF-κB, nuclear factor kappa B; JAK/STAT3, Janus kinase/signal transducer and activator of transcription 3; MAPK, mitogen activated protein kinase; PI3K/Akt, phosphatidylinositol 3 kinase/protein kinase B; Th, T helper; IL, interleukin; TNF-α, tumor necrosis factor alpha; OSA, obstructive sleep apnea. References: Liu et al., 2022; Chaput et al., 2022; St-Onge et al., 2019; Su et al., 2021; Dopytalska et al., 2020; Sáinz et al., 2014; Peng et al., 2021; Souza-Almeida et al., 2021; Yagishita et al., 2017; Kiełbowski et al., 2023; Johnston et al., 2008; Pan and Kastin, 2014; and Taheri et al., 2004. Created in BioRender. Amaral, L. (2026) https://BioRender.com/7mqtttz

Primarily produced by white adipose tissue, leptin is a hormone that regulates satiety and energy expenditure, and plays a central role in body metabolism [1]. It has a circadian secretion pattern [2], and its release peaks during the night, signaling the energy availability to the central nervous system and modulating the balance between hunger and satiety [2, 3]. Individuals with obesity develop resistance to this peptide, which means that even at elevated circulating levels, it cannot exert its expected effect, leading to persistent appetite and energy imbalance [1]. Modern lifestyle habits, represented by late-night and irregular meals, as well as insufficient sleep, can disrupt the alignment between endogenous circadian rhythms and environmental influence, changing the signaling of this adipokine [2, 4, 5]. While the metabolic effects of sleep disturbances on leptin regulation have been more clearly documented [2, 3], their repercussions in the skin are still unclear. Obesity, in turn, a chronic inflammatory and metabolic comorbidity, affects 19% of women and 14% of men worldwide; and can be associated with sleep disorders [4, 6]. With the increased global prevalence of this condition, research into the pathways shared with inflammatory dermatoses is essential to elucidate the metabolic impacts on the skin, including the role of leptin [4, 7]. This brief review proposes circadian leptin dysregulation as a potential upstream mechanism linking obesity, altered sleep and meal timing, oxidative stress, immune activation, and inflammatory skin diseases. By considering leptin rhythmicity as a mediator between chronobiology, metabolism, and dermatology, we present a testable hypothesis and highlight chronobiological strategies that may minimize cutaneous inflammation induced by obesity. The proposed circadian, metabolic, and cutaneous axis is summarized in Fig. 1. The main results are depicted in Table 1. To perform this review, a non-systematic literature search was conducted in PubMed using combinations of key terms: “leptin”, “obesity”, “circadian rhythm”, “sleep”, “sleep disorders”, “obstructive sleep apnea”, “meal timing”, “skin inflammation”, “psoriasis”, “hidradenitis *suppurativa*”, “oxidative stress”, and “inflammatory dermatoses”. The most relevant articles within this field were selected to support the proposed integrative framework.

### Circadian rhythm of leptin and central metabolic regulation

Physiologically, leptin exhibits biological oscillation, rising throughout the day and peaking at night [2, 8, 9]. This pattern inhibits appetite, optimizes energy metabolism, and supports tissue restoration processes. Leptin signaling is mainly mediated by the hypothalamus, regulating anorexigenic circuits with appetite reduction; and thermogenic circuits, that enhance heat production and energy consumption, aligning the balance between food intake and caloric expenditure [1]. The effectiveness of this hormone depends on the synchrony between circadian rhythms and eating behaviors, in which sleep regulation should be noted.

Sleep disruption can modify leptin homeostasis. Sleep restriction and deprivation can lead to reduced serum levels of this adipokine, inducing increased appetite and caloric intake, and greater preference for energy-dense foods. This occurs particularly during evening hours, when postprandial thermogenesis is lower, promoting positive energy balance and weight gain [4]. The overconsumption is a result of dysregulation of appetite hormones, suppression of leptin and elevation of ghrelin [4]. Beyond caloric excess, the timing of augmented food intake during circadian phases with low metabolic efficiency elevates metabolic burden [4]. The circadian misalignment of eating patterns impairs peripheral clock synchronization and appetite control mediated by leptin. This suggests that the weight-gain induction by sleep loss involves both quantitative and temporal components, represented, respectively, by greater intake and circadian desynchrony [10–12].

**Table 1:** Circadian and cutaneous effects of leptin in obesity

Aspects	Dysregulation	Molecular pathway	Clinical consequence	Therapeutic implication
Circadian leptin rhythm	Late meals, irregular eating patterns, and sleep disruption may alter leptin rhythmicity and favor leptin resistance [2, 4, 5]	Altered leptin signaling and hypothalamic appetite regulation [2]	Increased hunger, impaired satiety, weight gain, and systemic inflammation [17, 18]	Sleep regularity and meal timing alignment [4, 26]
Skin repair and epidermal homeostasis	Persistent hyperleptinemia may shift leptin signaling from reparative to pro-inflammatory activity [11, 15]	JAK/STAT3, MAPK, PI3K/Akt, angiogenic signaling [11, 23]	Keratinocyte activation, altered epidermal homeostasis, and impaired inflammatory control [11, 15]	Metabolic control and restoration of leptin sensitivity [11, 15]
Redox imbalance	Hyperleptinemia and metabolic or circadian disturbances may increase oxidative burden, while antioxidant capacity may be insufficient to counter persistent ROS generation [15, 19, 20]	ROS, redox-sensitive inflammatory signaling, and the KEAP1-NRF2 antioxidant response [19, 21]	Amplified inflammatory signaling, immune activation, and keratinocyte hyperproliferation (11, 20, 23]	Redox-targeted and NRF2-related approaches requiring further validation [20, 21]
Adipose–skin immune axis	Dysfunctional adipose tissue increases pro-inflammatory adipokines and reduces protective adipokines, such as adiponectin [11, 26]	Th1/Th17 polarization, IL-23/IL-17 axis, TNF-α, IL-6, endothelial activation [11, 23, 24]	Exacerbation of obesity-associated dermatoses, particularly psoriasis and hidradenitis *suppurativa *[15, 24, 25]	Weight management, metabolic optimization, and integrated dermatological and metabolic care [11, 15]

Notes: ROS, reactive oxygen species; NADPH oxidase, nicotinamide adenine dinucleotide phosphate oxidase; NRF2, nuclear factor erythroid 2-related factor 2; KEAP1, Kelch-like ECH-associated protein 1; NF-κB, nuclear factor kappa B; JAK/STAT3, janus kinase/signal transducer and activator of transcription 3; MAPK, mitogen-activated protein kinase; PI3K/Akt, phosphatidylinositol-3-kinase/protein kinase B; Th, T helper; IL, interleukin; TNF-α, tumor necrosis factor alpha

Fragmented sleep is a characteristic of obstructive sleep apnea (OSA), a prevalent and multisystemic sleep disorder that can be related to obesity, and is often accompanied by hyperleptinemia [3]. This elevated levels can be a result from both obesity and neuroendocrine dysregulation induced by intermittent hypoxia and chronic inflammation observed in OSA [3, 8]. In this context, persistently augmented concentrations of leptin reflect central resistance to this hormone, generating energy imbalance [8, 11, 12]. 

#### Influence of meal timing on leptin

The circadian oscillation of leptin depends on the endogenous clock, and is influenced by the meal times. Changes in the eating cycle can alter the serum values of leptin, even in the absence of sleep imbalance. The effectiveness of leptin signaling results from the synchrony between the circadian rhythm and lifestyle habits, and its disruption can compromise metabolic regulation and promote hormone resistance [3]. 

A controlled clinical study considering normal and late schedules for sleep and meal-timing in adults with overweight or obesity has investigated their influence on food intake, regardless of sleep duration. Hormones connected to appetite were evaluated, including leptin, ghrelin, and glucagon-like peptide-1 (GLP-1). Considering constant sleep duration, the study found a significant connection between sleep and meal timing, which may influence energy intake and balance. The leptin concentrations at night have been encountered elevated in the cases of regular meal habits. In response to the meal tolerance test, ghrelin and GLP-1 presented significant interaction with sleep-meal timing [5]. These findings suggest that the balance between sleep and meals may influence energy patterns and intake, as well as hormonal regulation of appetite; although specific effects, particularly for leptin, should be carefully interpreted.

Late meal times compromise leptin signaling and can lead to higher caloric intake and less healthy food choices. This effect can be exacerbated in obese individuals with hyperleptinemia [1, 10]. In a population-based sleep study, sleeping less than 5h per night, according to polysomnography registers, was associated with a 15.5% reduction in fasting leptin; and 14.9% elevation in ghrelin levels compared with 8h of sleep, independently of body mass index (BMI) [13]. These data should be inferred as a correlation with habitual short sleep rather than as a direct effect of acute sleep deprivation. In contrast, obesity and OSA are commonly related to hyperleptinemia, reflecting impaired leptin sensitivity and contributing to chronic metabolic inflammation [3, 14, 15]. A systematic review of sleep parameters in adolescents highlighted that insufficient sleep duration and poor sleep quality were correlated to reduced leptin levels and increased inflammation [15], a scenario that may exacerbate skin complications in obesity [16]. 

Leptin resistance in obesity should not be regarded as a loss of leptin action. It may represent a selective and tissue-specific process [11, 12]. Central leptin resistance refers to impaired hypothalamic responses to this hormone, resulting in a diminished anorexigenic response, persistent appetite, and body weight regulation despite hyperleptinemia [11]. In contrast, leptin signaling may remain active in peripheral tissues and immune cells [12, 17]. Experimental evidence in diet-induced obesity has pointed that leptin may fail to reduce food intake, indicating central resistance, while still inducing peripheral inflammatory responses, including tumor necrosis factor alpha (TNF-α) production, neutrophil recruitment, and macrophage activation [17]. Hyperleptinemia may coexist with impaired central metabolic control and preserved peripheral pro-inflammatory signaling [11, 17]. Leptin resistance can contribute to obesity, whereas elevated leptin levels may amplify immune activation and inflammatory pathways relevant to obesity-associated dermatoses [11, 12, 17]. 

## Leptin, skin, and inflammation in obesity: influences of sleep

The skin expresses leptin receptors, particularly in keratinocytes, fibroblasts, endothelial cells, and follicular structures. Given its pleiotropic functions, leptin can participate in the modulation of angiogenesis, lipid metabolism, and immune response [16]. In healing processes, this peptide can stimulate the migration and proliferation of keratinocytes and fibroblasts, the formation of new vessels and collagen synthesis, which accelerates re-epithelialization and optimize tissue repair. After a local injury, leptin action in the skin can increase, contributing to a faster repair response. Regarding immunity, this hormone defends skin tissue against microorganisms by inducing the production of antimicrobial peptides, like beta-defensin 2, strengthening the immune barrier of the epidermis [8]. 

In sleep disturbances, particularly OSA, leptin dysregulation, metabolic imbalance, and cutaneous inflammation may clinically converge [1, 3, 18, 19]. OSA is frequently related to obesity and is characterized by sleep fragmentation and intermittent hypoxia, both of which can promote oxidative stress, endothelial dysfunction, sympathetic activation, and low-grade systemic inflammation [14, 18]. Elevated leptin levels in obesity can trigger a pro-inflammatory response. Hyperleptinemia can be a result of increased adiposity and impaired leptin sensitivity, and intermittent hypoxia may further amplify reactive oxygen species (ROS) generation and inflammatory pathways [1, 3, 18]. Its excessive concentration activates intracellular pathways, markedly the janus kinase/signal transducer and activator of transcription 3 (JAK/STAT3), the mitogen-activated protein kinase (MAPK), and the phosphatidylinositol-3-kinase/protein kinase B (PI3K/Akt). The production of inflammatory cytokines can be augmented, encompassing the interleukin (IL)-1, IL-6, IL-17, IL-23, and TNF-α, as well as increasing the release of ROS, which contributes to chronic skin inflammation [8]. 

The direct clinical evidence connecting OSA-related leptin changes to inflammatory skin disorders remains limited. Several pathways, involving oxidative stress, ROS generation, cytokine release, and T helper (Th)1/Th17 polarization, may overlap with mechanisms implicated in inflammatory dermatoses linked to obesity, like psoriasis and hidradenitis *suppurativa* [1, 3, 8, 16, 18]. Endothelial disruption can occur as a result of inflammation and hypoxemia related to OSA; this scenario leads to a pro-inflammatory endothelial state that facilitates leukocyte adhesion and recruitment, as well as altered cellular perfusion. Sleep disorders, particularly OSA, may represent a translational model in further research for investigating whether improving sleep quality, reducing intermittent hypoxia, and restoring leptin sensitivity and metabolic regulation can attenuate inflammatory exacerbation in obesity interrelated dermatoses [1, 3, 18]. 

### Leptin and redox imbalance in skin inflammation

An imbalance between ROS generation and antioxidant defense may link leptin dysregulation to cutaneous inflammation. In human keratinocytes, leptin increased ROS generation and altered the cellular redox state, with adaptive antioxidant responses including catalase induction and enhanced dehydroascorbate reduction [20]. Evidence from OSA and obesity has correlated leptin resistance and hyperleptinemia with oxidative stress [21]. In psoriasis, oxidative stress is more clearly established; ROS can damage cellular macromolecules and activate inflammatory pathways, contributing to cytokine induced inflammation and keratinocyte hyperproliferation [22]. This redox inflammatory relation is clinically relevant, as individuals with psoriasis present higher ROS production and reduced antioxidative capacity [22]. The proposed sequence is that leptin dysregulation may increase oxidative burden; this insufficient antioxidant control may amplify inflammatory signaling; and immune activation may contribute to cutaneous inflammation [2, 17, 21, 22]. NRF2 should be raised as part of this antioxidant response. Experimental evidence indicated that NRF2 dependent expression regulates epidermal differentiation and limits psoriasiform inflammation. In NRF2-deficient mice, imiquimod-induced skin inflammation was exacerbated, while NRF2 activation attenuated psoriasiform reactions and supported epidermal homeostasis [22, 23]. Outside the skin, NRF2 activity reduced hypothalamic oxidative stress and improved leptin responsiveness in experimental models [24]. Direct evidence linking leptin dysregulation to impaired NRF2 signaling in human inflammatory dermatoses remains limited.

Against this mechanistic background, the relationship between leptin and inflammatory skin disorders appears to be disease specific rather than uniform across conditions. Psoriasis is a relevant model because of its association with obesity, metabolic burden, systemic inflammation, keratinocyte hyperproliferation, and activation of the Th1/Th17 and IL-23/IL-17 inflammatory axes [8, 16, 25]. A study demonstrated that leptin increased the release of IL-1β in blood monocytes, while significantly inducing the secretion of the growth factor amphiregulin in ex-vivo psoriatic skin. These findings suggest that leptin acts as a bridge between adipose tissue dysfunction, systemic pro-inflammatory cytokine production, and local keratinocyte proliferation [26]. Obese patients with psoriasis have presented higher leptin serum levels and increased leptin mRNA expression in subcutaneous adipose tissue, correlated with psoriasis severity [27]. These data support the concept of an adipose and skin axis, in which impaired adipose tissue may contribute to cutaneous inflammation through adipokine imbalance, cytokine production, oxidative stress, endothelial activation, and immune imbalance. Hidradenitis *suppurativa* is another skin disorder connected to obesity, follicular inflammation, and immune activation. Other conditions, like atopic dermatitis, cutaneous lupus erythematosus, and scleroderma have heterogeneous associations with leptin, which may reflect modifying effects of this hormone instead of a possible direct role in disease triggering [8, 16]. 

In addition to leptin dysregulation, obesity is characterized by reduced adiponectin, an adipokine with anti-inflammatory and metabolic regulatory properties that can be influenced by circadian regulation [9]. In systemic sclerosis, lower adiponectin pathway activity has been associated with skin fibrosis, while experimental studies indicate anti-fibrotic effects of adiponectin in dermal fibroblasts, including AMPK activation [28]. Separately, JAK2/STAT3 signaling has a pro-fibrotic role in systemic sclerosis fibroblasts, promoting myofibroblast differentiation, collagen release, and fibrosis [29]. Leptin has immunomodulatory effects relevant to inflammatory skin disease and can activate JAK2/STAT3 signaling in responsive cells [8, 25]. However, clinical studies of circulating leptin in systemic sclerosis have produced conflicting findings [30, 31]. It has not yet been fully established whether lower leptin levels directly inhibit JAK/STAT3 signaling in systemic sclerosis.

Psoriasis and hidradenitis *suppurativa* may be the most suitable clinical models for investigating circadian leptin dysregulation in inflammatory skin diseases in the context of obesity. This comorbidity aggravates the skin response, favoring a higher prevalence of dermatoses related to chronic inflammation. The association between sleep disorders and obesity exacerbates this scenario, suggesting that the skin is not only a passive target organ, but an integral part of the consequences of metabolic disruption [4, 7]. 

## Chronobiological interventions: evidence from intermittent fasting and time-restricted eating

Synchronizing eating habits with circadian rhythms is an innovative and underexplored approach in dermatology, especially in the context of excessive adiposity and skin complications. This perspective is currently gaining prominence due to growing interest in chronobiology-based therapies. It offers new possibilities for reducing systemic pro-inflammatory states and their manifestation on the skin. Studies have investigated strategies, notably intermittent fasting (IF), which alternates periods of eating with prolonged fasting; and time-restricted feeding (TRF), that limits caloric intake to a specific daily window (e.g., 8–10h). This points out their potential to modulate inflammatory physiology in obese individuals.

A systematic review and meta-analysis enrolling overweight or obese adults, compared IF and TRF with controls. The results demonstrated significant reductions in the inflammatory markers IL-6, TNF-α, and C-reactive protein (CRP), with emphasis on the skin. Both IF and TRF were more effective in reducing chronic inflammation than caloric restriction alone [32]. These markers may be considered as targets in skin diseases, suggesting that IF and TRF may attenuate inflammatory pathways regulated by leptin in the skin, improving tissue repair and reducing hyperleptinemia.

The impact of the type of diet is not limited to reducing inflammation. Early time-restricted feeding (eTRF), in which food intake is concentrated earlier in the day, has been associated with changes in the expression of selected circadian clock genes measured in whole blood cells. In a study, eTRF increased morning expression of *BMAL1*, *CRY1*, *CRY2*, and *RORA*, decreased evening expression of *PER1*, and increased evening expression of *CRY1*, *CRY2*, and *RORA*, whereas *CLOCK* and *PER2* were not significantly affected [33]. These findings suggest that early meal timing may influence peripheral circadian clock markers, but further studies are needed to determine whether these changes reflect altered rhythmicity, amplitude, or phase alignment [33]. The relationship between sleep total time, leptin, and inflammation depends on the metabolic context. Short sleep duration and experimental sleep loss have been correlated with lower circulating leptin, higher ghrelin, increased hunger, and changes in inflammatory mediators, such as IL-6 and TNF-α [18, 19]. This pattern does not necessarily conflict with the hyperleptinemia observed in obesity. In short-term sleep restriction, lower leptin may favor appetite stimulation and subsequent increases in energy intake, whereas chronic obesity is characterized by increased adipose mass, persistently elevated leptin levels, and leptin resistance [18, 19]. 

Sleep disruption may contribute to the metabolic and inflammatory imbalance associated with obesity. Precise estimates of inflammatory skin disease among patients with both obesity and sleep disorders are still limited, although sleep disturbances and circadian misalignment have been associated with increased obesity risk [4, 13–15]. This interaction is partly explained by metabolic and hormonal changes induced by insufficient sleep, encompassing altered appetite hormone regulation, changes in food intake, and leptin imbalance [2, 4, 13, 18, 21]. However, sleep, obesity, leptin regulation, and inflammatory dermatoses are usually studied separately, and integrated epidemiological data remain scarce [1, 3, 8, 16]. 

Melatonin, an indoleamine secreted by the pineal gland, may be a strategy for modulating the circadian rhythms of adipokines, including adiponectin. In a pre-clinical study, melatonin supplementation in rats at a dose of 10 mg/kg/day for 6 weeks promoted the conversion of inguinal white adipose tissue toward a brown-like phenotype, suggesting its potential role in adipose tissue remodeling and metabolic regulation [34]. These effects may be relevant to the metabolic and inflammatory repercussions of obesity. In humans, melatonin supplements can modulate circadian rhythms; however, doses and responses observed in animal models require clinical validation to confirm their metabolic and anti-inflammatory relevance. Melatonin supplementation may have a potential part in modulating adipokine secretion patterns and systemic inflammation [35]. This could indirectly influence skin complications associated with obesity, but it requires validation in cases of inflammatory dermatoses in humans. These findings may be a basis for future investigations that integrate these benefits into therapeutic protocols.

Although leptin may contribute to skin inflammation in the context of obesity and sleep disruption, the available evidence primarily supports a biologically plausible association. Additional mechanistic and interventional studies are needed to establish causality and validate the proposed relationships.

## Conclusions

This review proposes circadian leptin dysregulation as a potential link between obesity, sleep disruption, altered meal timing, redox imbalance, immune activation, and inflammatory skin disease. In this model, circadian and metabolic disturbances may alter leptin signaling and increase oxidative burden; when antioxidant defenses are insufficient, redox sensitive inflammatory pathways may be amplified and contribute to cutaneous inflammation. Current evidence remains largely associative, and leptin should be considered as a potential inflammatory mediator rather than an independent cause of dermatological disease. Longitudinal and interventional studies are needed to determine whether leptin dysregulation directly influences the development or progression of inflammatory dermatoses associated with obesity. Chronobiological approaches, including TRF and interventions that improve sleep regularity, warrant further study for their effects on leptin regulation and skin inflammation. Future trials should evaluate these strategies in populations with obesity and inflammatory skin disease and determine whether changes in leptin signaling, oxidative stress, and inflammatory pathways accompany clinical outcomes.

## Data Availability

No datasets were generated or analysed during the current study.
